# Data on different types of green spaces and their accessibility in the seven largest urban regions in Finland

**DOI:** 10.1016/j.dib.2023.109458

**Published:** 2023-07-31

**Authors:** Vuokko Heikinheimo, Maija Tiitu, Arto Viinikka

**Affiliations:** Finnish Environment Institute Syke, Latokartanonkaari 11, 00790 Helsinki, Finland

**Keywords:** Urban green space, Green space quality, Spatial accessibility, Network distances, Environmental equity, Open data

## Abstract

Access to green spaces in urban regions is vital for the well-being of citizens. In this article, we present data on green space quality and path distances to different types of green spaces. The path distances represent green space accessibility using active travel modes (walking, cycling). The path distances were calculated using the pedestrian street network across the seven largest urban regions in Finland. We derived the green space typology from the Urban Atlas Data that is available across functional urban areas in Europe and enhanced it with national data on water bodies, conservation areas and recreational facilities and routes from Finland. We extracted the walkable street network from OpenStreetMap and calculated shortest paths to different types of green spaces using open-source Python programming tools. Network distances were calculated up to ten kilometers from each green space edge and the distances were aggregated into a 250 m × 250 m statistical grid that is interoperable with various statistical data from Finland. The geospatial data files representing the different types of green spaces, network distances across the seven urban regions, as well as the processing and analysis scripts are shared in an open repository. These data offer actionable information about green space accessibility in Finnish urban regions and support the integration of green space quality and active travel modes into further research and planning activities.

**Specifications Table**

**Table:** 

Subject	Geography
Specific subject area	Green space quality and spatial accessibility
Type of data	Table Spatial database (GeoPackage) Figures
How the data were acquired	The green space typology was derived from Urban Atlas data [1] and further modified using ancillary data on water bodies and conservation areas [2], [3], [4], and recreation facilities and routes from the Lipas sport facility GIS database [5]. The street network data were acquired from OpenStreetMap (OSM) downloaded in Protocolbuffer Binary Format (PBF) from https://www.geofabrik.de/[6]. Network distances were calculated based on OSM pedestrian street network. Data acquisition and network analysis were conducted using the Python programming language (version 3.8) and standard geospatial Python libraries such as geopandas (version 0.8.2). Pyrosm library (version 0.6.1) was used for fetching and cropping the network data, and Pandana library (version 0.6) was used for calculating network distances to nearest green spaces across the study regions.
Data format	Raw Analyzed
Description of data collection	Green space data were compiled for seven largest urban regions in Finland. Green space edges were converted into a set of points to serve as potential access points. Network analysis was conducted from each node in the walkable network to network nodes associated with green space edges. If origin location was inside green space, then distance was set to 0 m. If distance was 10,000 m or greater, distance was set to NULL. Final values were averaged into a 250 m x 250 m statistical grid.
Data source location	­ Raw data for green areas: Urban Atlas [1] ­ Raw data for water bodies: Shoreline10 [2] and Shoreline250 [3] ­ Raw data for conservation areas: The nature protected areas and wilderness reserves dataset [4] ­ Raw data for facilities and routes: LIPAS database [5] ­ Raw data for pedestrian network: OpenStreetMap [6] ­ Urban core areas: continuous inner and outer urban areas combined as delineated in urban–rural classification [7] ­ Statistical grid: 250 m x 250 m grid [8] ­ Raw data for building locations: Building and Dwelling Register [9] ­ Functional Urban Regions in Finland with more than 100,000 inhabitants: Helsinki, Jyväskylä, Kuopio, Lahti, Oulu, Tampere and Turku: Urban Atlas [1]
Data accessibility	Repository name: Zenodo Data identification number: 10.5281/zenodo.8091921 Direct URL to data: https://doi.org/10.5281/zenodo.8091921
Related research article	A. Viinikka, M. Tiitu, V. Heikinheimo, J.I. Halonen, E. Nyberg, K. Vierikko, Associations of neighborhood-level socioeconomic status, accessibility, and quality of green spaces in Finnish urban regions, Appl. Geogr. 157 (2023) 102973. https://doi.org/10.1016/j.apgeog.2023.102973.

## Value of the Data

• Data on green space quality and accessibility covering largest urban regions in Finland allows regional comparisons.
• These data allow researchers, local planners and decision-makers to investigate the spatial accessibility and quality of green spaces.
• The data can support impact assessments of land use development options and help to avoid fragmenting and reducing the diversity of green spaces.
• Information about green space quality helps to integrate varying perspectives of green space use from citizens’ point of view into further assessments.
• The accessibility data are interoperable with various statistical data from Finland.
• Open method and open data allow reproducibility over time and beyond the study area.

## 1 Objective

The objective of this article is to provide a detailed description of publicly shared data on green space quality and green space accessibility from the seven largest urban regions in Finland. This data article complements a related research article where these data were used to investigate associations of neighborhood-level socio-economic status, accessibility, and quality of green spaces. This data article adds value to the original research article by providing additional details of the green space quality and accessibility data and the underlying methodology and assumptions. The described data are interoperable with various statistical data in Finland and this data article supports the use of these data in further analyses of equal access to different types of green spaces.

## 2 Data Description

This article describes data used in Viinikka et al. [10] to assess green space quality and accessibility across the seven largest urban regions in Finland. Data related to this article are shared openly in a data repository [11]. Text file “README.txt” contains a short description of the data and available attributes. Spatial data are stored in GeoPackage format which is an open format for geospatial information. The coordinate reference system of all spatial data is ETRS-TM35FIN (EPSG:3067). The layers can be viewed, for example, in the QGIS software. The QGIS project file “green_space_quality_and_accessibility.qgz” contains the data described in this article visualized in thematic layers.

### 2.1 Green Space Quality Data

**Fig. 1:**
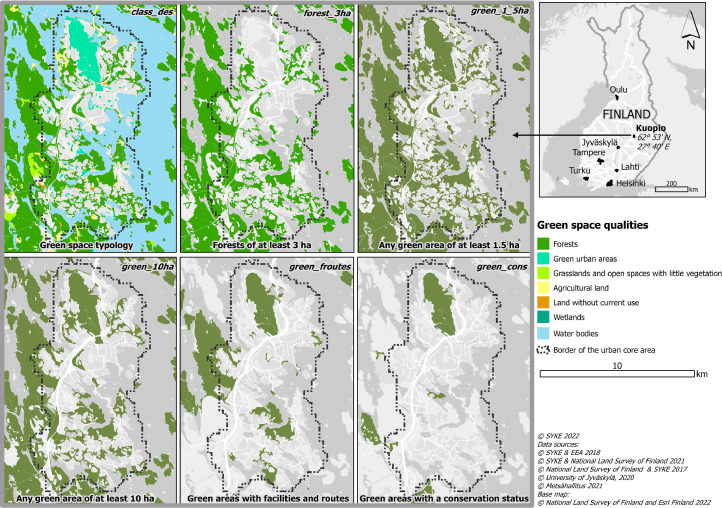
Green space quality data exemplified in the Kuopio urban region. (For interpretation of the references to color in this figure legend, the reader is referred to the web version of this article.)

The file “green_spaces.gpkg” contains green space polygons for the seven Finnish urban regions with several attributes to describe the quality of the green space. Table 1 contains a description of the data attributes, and Fig. 1 presents example visualizations of different green space quality classes. Water bodies are provided as a separate file “water_bodies.gpkg”. The column ‘centr_id’ contains the unique identifier for each data feature, and it is formed based on the x and y coordinates of the centroid of each polygon. The columns ‘region’ and ‘core’ describe which urban region the polygon belongs to and whether it is part of the urban core area or not. It is possible to investigate only the green spaces that are located among the dense urban structure, or only the more sparsely built peri-urban areas by filtering the data based on the ‘core’ column.

**Table 1:** Attributes in the green space data.

Column name	Description
centr_id	Unique identifier of the green space polygons. 6 first digits represent the x-coordinate, 7 last digits represent the y-coordinate of the polygon centroid in ETRS-TM35FIN.
region	Name of the functional urban area
core	Whether the centroid of the green space belongs to the urban core area (1 = yes, 0 = no)
area	The total area of the green space in hectares (ha)
class	Land cover typology of the green space (A–F)
class_des	Description of the green space land cover categories: A = Forests B = Green urban areas C = Grasslands and open spaces with little vegetation D = Agricultural land E = Wetlands F = Land without current use G = Water bodies
forest_3 ha	Whether the green space is a forest with a minimum size of 3 ha (1 = yes, 0 = no)
forgua_1_5 ha	Whether the green space is a forest or a green urban area with a minimum size of 1.5 ha (1 = yes, 0 = no)
forgua_3 ha	Whether the green space is a forest or a green urban area with a minimum size of 3 ha (1 = yes, 0 = no)
green_1_5 ha	Whether the green space (all categories) has a minimum size of 1.5 ha (1 = yes, 0 = no)
green_3 ha	Whether the green space (all categories) has a minimum size of 3 ha (1 = yes, 0 = no)
green_5 ha	Whether the green space (all categories) has a minimum size of 5 ha (1 = yes, 0 = no)
green_10ha	Whether the green space (all categories) has a minimum size of 10 ha (1 = yes, 0 = no)
green_cons	Whether the green space has a conservation status: at least 25% of the patch area is conserved (1 = yes, 0 = no)
green_facilities	Whether the green space includes at least one facility (1 = yes, 0 = no)
green_routes	Whether the green space includes at least one route (1 = yes, 0 = no)
green_froutes	Whether the green space includes at least one facility and route (1 = yes, 0 = no)

The columns ‘class’ and ‘class_des’, adapted from the original Urban Atlas data, differentiate between the land cover categories that are represented in the green space data: forests, green urban areas, grasslands and open spaces with little vegetation, agricultural land, land without current use, and wetlands. Water bodies were derived from national datasets (Shoreline10 and Shoreline250). Water dataset includes only a subset of the columns; ‘centr_id’, ‘region’, ‘class’ (“G”) and ’class_des’ (“Water bodies”).

Several columns regarding the land cover and size of the green spaces have been calculated based on information in columns ‘class’ and ‘area’. There is information on whether the feature belongs to class “forest” with a minimum size of 3 ha (‘forest_3 ha’), “forest” or “green urban area” with a minimum size of 1.5 (‘forgua_1_5 ha’) or 3 ha (‘forgua_3 ha’), or whether the feature is any green space with minimum sizes of 1.5, 3, 5, and 10 ha (‘green_1_5_ha’, ‘green_3 ha’, ‘green_5 ha’, ‘green_10ha’). In addition, the data contains information on whether at least 25% of the area of the green space is conserved (‘green_cons’), and whether the green space contains recreational facilities (‘green facilities’), routes (‘green_routes’) or both (‘green_froutes’) based on the Lipas sport facility database [5].

### 2.2 Accessibility Data

**Fig. 2:**
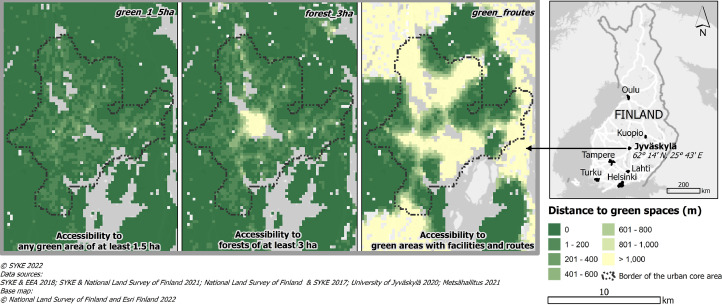
Green space accessibility data exemplified in the Jyväskylä urban region. (For interpretation of the references to color in this figure legend, the reader is referred to the web version of this article.)

The file “green_space_accessibility.gpkg” contains the network distances to different types of green spaces from all of the seven urban regions. See example visualization in Fig. 2. The data are also available as a CSV (text file with comma-separated values) “green_space_accessibility.csv”. Table structure is identical in the GeoPackage file and the CSV file with the exception that the GeoPackage contains geographic coordinates. Column names and their descriptions are detailed in Table 2. The first two columns, ‘xyind’ and ‘region’ are spatial identifiers. The column ‘ykrid’ is also the unique identifier for each grid square. The column ‘region’ contains the name of the functional urban area and the data can be split per region based on this column. The rest of the columns represent distances in the pedestrian street network to different types of green and blue spaces. Distances are in meters (m) and have been rounded to the nearest meter. Value range of the distance values is from zero to 9,999 meters. Distance is zero, if the grid square centroid is in the target green space. Distance is NULL if the path distance is >=10,000 meters, or if the grid square does not overlap with the pedestrian street network. This threshold was chosen to allow longer distances to more sparsely located green space types (such as those with conservation status) while maintaining the focus in neighborhood-level analysis within the range of typical walking and cycling trip lengths.

**Table 2:** Attributes in the green space accessibility data.

Column name	Description
xyind	Unique identifier of the statistical grid square. 6 first digits represent the x-coordinate, 7 last digits represent the y-coordinate of the grid square centroid in the ETRS-TM35FIN coordinate reference system
region	Name of the functional urban area
core	Whether the grid square belongs to the urban core area (1 = yes, 0 = no)
green_all	Path distance (m) to closest green space (all categories)
gua	Path distance (m) to closest green urban area
forest	Path distance (m) to closest forest
forest_3 ha	Path distance to closest forest, minimum size 3 ha
forgua	Path distance (m) to closest forest or green urban area
forgua_1_5 ha	Path distance (m) to closest forest or green urban area, minimum size 1.5 ha
forgua_3 ha	Path distance (m) to closest forest or green urban area, minimum size 3 ha
green_1_5 ha	Path distance (m)to closest green space (all categories, minimum size 1.5 ha)
green_3 ha	Path distance (m) to closest green space (all categories, minimum size 3 ha)
green_5 ha	Path distance (m) to closest green space (all categories, minimum size ha)
green_10 haha	Path distance (m) to closest green space (all categories, minimum size 10 ha)
green_cons	Path distance (m) to closest green space with conservation status (at least 25% of the patch area is conserved)
green_facilities	Path distance (m) to closest green space that includes at least one facility
green_routes	Path distance (m) to closest green space that includes at least one route
green_froutes	Path distance (m) to closest green space that includes at least one facility and route
water	Path distance (m) to closest sea, lake or river with a minimum diameter of 5 m

## 3 Experimental Design, Materials and Methods

### 3.1 Green Space Quality Attributes

The first step of building the green space dataset was to extract the land cover categories to be considered as green spaces. To select the appropriate classes, we used Urban Atlas data mapping guide [12], definitions for urban green infrastructure by Maes et al. [13] and spatial comparison of the Urban Atlas data and orthophotos in Finnish urban regions. The Urban Atlas mapping guide separates classes on land as artificial surfaces, agricultural, natural and semi-natural areas, and wetlands [12]. Self-evidently, we classified all classes belonging to natural and seminatural areas and wetlands as green spaces. Also, all the agricultural classes were included, since in Finnish context, those areas are widely used for recreation, especially in the wintertime. Of the artificial surfaces, we defined the classes “green urban areas” and “land without current use” as green spaces. “Green urban areas” contained the main public recreation areas inside urban areas, and “land without current use” was included, since the areas belonging to the class in Finnish context contained a lot of vegetated empty lots among residential areas that we hypothesized as potentially important for urban biodiversity. In contrast, the artificial classes “discontinuous low density urban fabric” and “discontinuous very low density urban fabric” were not included, since despite the low degree of soil sealing (<30%) of the classes [12], the green spaces in this type of residential areas can be assumed to be mostly private gardens not publicly accessible.

Urban Atlas data was reclassified to optimally identify the diverse types of green spaces in Finland. The classes “forests”, “green urban areas”, “wetlands”, and ”land without current use” were directly retrieved from the Urban Atlas source data. The classes “grasslands and open spaces with little vegetation” is a combination of two Urban Atlas classes: “herbaceous vegetation associations (natural grasslands, moors)” and “open spaces with little or no vegetation (beaches, dunes, bare rocks, glaciers)”. The class “agricultural land” contains the Urban Atlas classes “arable land” and “pastures”. The source data polygons of Urban Atlas were dissolved according to this classification, meaning that the adjacent polygons belonging to the same land cover categories were merged into one polygon.

We used ArcMap (version 10.8.1) tool Tabulate area to first calculate the acreage of nature conservation areas (m^2^) [4] in each green space polygon. Then we calculated the percentage of conservation areas in each green space polygon by dividing the conserved area by the total acreage of the green space. Based the authors’ expert evaluation and local knowledge on different cities, the green spaces containing at least 25% of conserved area were classified as green spaces with conservation status. We also analyzed the existence of recreational facilities and existence of recreational routes based on spatial overlap using the LIPAS database [5]. Water bodies were extracted from two national databases: Shoreline10 [2] for seas and lakes and Shoreline250 [3] for rivers.

### 3.2 Network Analysis

Green space and water body edges were converted into a set of points at ten-meter interval to serve as potential access points in the network analysis. We calculated distances to different types of green spaces from each node of the walkable street network separately for each urban region. Walkable paths were extracted using the Pyrosm Python library (version 0.6.1; setting network_type=”walking” in the get_network -method). Network analysis was implemented using the Pandana Python library, which applies contraction hierarchies to calculate shortest paths in a speeded-up manner [14].

Green space edge points were connected to the walkable network (using the set_pois method in Pandana). This way, the analysis allowed access to green spaces from any direction; the routing stopped at the network node located nearby a green space edge. Then, the distance from each node in the network to the nearest green space node was calculated (using the nearest_pois method in Pandana). Path distances to nearest green space edge were calculated up to 10,000 meters. If distance was 10,000 m or greater, the value was set to NULL. For locations inside the target green space type, then distance was set to 0 m. The network analysis was repeated separately for each urban green space quality class.

Finally, the path distances were averaged into the 250 m × 250 m statistical grid squares. The averaging was done separately for inhabited and uninhabited grid squares. As an intermediate step, we attached the path distances to the locations of inhabited buildings [9]. For grid squares containing inhabited buildings, the final value was calculated as the average of the street nodes closest to each inhabited building. For grid squares outside inhabited areas, the final value was calculated as the mean value of all street nodes within that grid square.

Quality of the data set is subject to the quality of the underlying data sets. Each source data have their distinct sources of error and uncertainty. Overall, used data sets represent the best available regional data on green spaces [1], water bodies [2,3], recreational facilities [5] and pedestrian street network [6] across the Finnish urban regions.

## Ethics Statements

The work meets ethical policies and quidelines of academic publishing. No human subjects, animal experiments or any data collected from social media platforms were included in this work.

## CRediT authorship contribution statement

**Vuokko Heikinheimo:** Conceptualization, Methodology, Software, Data curation, Formal analysis, Investigation, Validation, Writing – original draft, Writing – review & editing. **Maija Tiitu:** Conceptualization, Methodology, Validation, Formal analysis, Investigation, Data curation, Writing – original draft, Writing – review & editing, Visualization, Funding acquisition. **Arto Viinikka:** Conceptualization, Validation, Writing – review & editing, Project administration, Funding acquisition.

## Data Availability

Data on different types of green spaces and their accessibility in the seven largest urban regions in Finland (Original data) (Zenodo).

## References

[1] Finnish environment institute (Syke), European Environment Agency (EEA), Urban atlas, 2018. https://ckan.ymparisto.fi/dataset/kaupunkiatlas-urban-atlas (accessed May 25, 2022).

[2] Finnish environment institute (Syke), National Land Survey of Finland (NLS), Shoreline10 (Fin: Ranta10 - rantaviiva 1:10 000), 2021. https://ckan.ymparisto.fi/dataset/ranta10-rantaviiva-1-10-000 (accessed May 25, 2022).

[3] National Land Survey of Finland (NLS), Finnish environment institute (Syke), Shoreline250 (Fin: Ranta250 - rantaviiva 1: 250 000), 2017. https://ckan.ymparisto.fi/dataset/ranta250-rantaviiva-1-250-000.

[4] Metsähallitus, The nature protected areas and wilderness reserves dataset (Fin: Luonnonsuojelu- ja erämaa-alueet), 2021. https://ckan.ymparisto.fi/dataset/luonnonsuojelu-ja-eramaa-alueet (accessed May 25, 2022).

[5] University of Jyväskylä, Lipas sport facility GIS-database, 2020. https://www.jyu.fi/sport/en/cooperation/lipas/lipas-system (accessed March 1, 2020).

[6] OpenStreetMap contributors, OpenStreetMap, 2021. https://www.geofabrik.de/data/download.html (accessed May 25, 2022).

[7] Finnish environment institute (Syke), YKR Urban-rural classification (Fin: YKR Kaupunki-maaseutu-luokitus), 2020. https://ckan.ymparisto.fi/dataset/kaupunki-maaseutu-luokitus-ykr (accessed May 25, 2022).

[8] Statistics Finland, Finnish environment institute (Syke), 250 m x 250 m statistical grid, 2021.

[9] Digital and Population Data Services Agency, Building and Dwelling Register (Fin: Väestötietojärjestelmän rakennus- ja huoneistotiedot RHR), 2021. https://ckan.ymparisto.fi/en/dataset/vaestotietojarjestelman-rakennus-ja-huoneistotiedot-rhr (accessed May 25, 2022).

[10] Viinikka A., Tiitu M., Heikinheimo V., Halonen J.I., Nyberg E., Vierikko K. (2023). Associations of neighborhood-level socioeconomic status, accessibility, and quality of green spaces in Finnish urban regions. Appl. Geogr..

[11] Heikinheimo V., Tiitu M., Viinikka A. (2023). Data on different types of green spaces and their accessibility in the seven largest urban regions in Finland. Zenodo.

[12] European Comission, Mapping Guide v6.2 for a European Urban Atlas., 2020. https://land.copernicus.eu/user-corner/technical-library/urban_atlas_2012_2018_mapping_guide (accessed June 20, 2023).

[13] J. Maes, G. Zulian, S. Guenther, M. Thijssen, J. Raynal, Enhancing resilience of urban ecosystems through green infrastructure (EnRoute), JRC115375 (2019). doi:10.2760/689989.

[14] Foti F., Waddell P., Luxen D. (2012). Proc. 4th TRB Conf. Innov. Travel Model.

